# Multimodality Advanced Cardiovascular and Molecular Imaging for Early Detection and Monitoring of Cancer Therapy-Associated Cardiotoxicity and the Role of Artificial Intelligence and Big Data

**DOI:** 10.3389/fcvm.2022.829553

**Published:** 2022-03-15

**Authors:** Jennifer M. Kwan, Evangelos K. Oikonomou, Mariana L. Henry, Albert J. Sinusas

**Affiliations:** ^1^Section of Cardiovascular Medicine, Yale University School of Medicine, New Haven, CT, United States; ^2^Geisel School of Medicine at Dartmouth, Hanover, NH, United States; ^3^Department of Radiology and Biomedical Imaging, Yale University School of Medicine, New Haven, CT, United States; ^4^Department of Biomedical Engineering, Yale University, New Haven, CT, United States

**Keywords:** cardiotoxicity, cardiovascular imaging, big data, cancer therapy-associated cardiotoxicity, molecular imaging

## Abstract

Cancer mortality has improved due to earlier detection *via* screening, as well as due to novel cancer therapies such as tyrosine kinase inhibitors and immune checkpoint inhibitions. However, similarly to older cancer therapies such as anthracyclines, these therapies have also been documented to cause cardiotoxic events including cardiomyopathy, myocardial infarction, myocarditis, arrhythmia, hypertension, and thrombosis. Imaging modalities such as echocardiography and magnetic resonance imaging (MRI) are critical in monitoring and evaluating for cardiotoxicity from these treatments, as well as in providing information for the assessment of function and wall motion abnormalities. MRI also allows for additional tissue characterization using T1, T2, extracellular volume (ECV), and delayed gadolinium enhancement (DGE) assessment. Furthermore, emerging technologies may be able to assist with these efforts. Nuclear imaging using targeted radiotracers, some of which are already clinically used, may have more specificity and help provide information on the mechanisms of cardiotoxicity, including in anthracycline mediated cardiomyopathy and checkpoint inhibitor myocarditis. Hyperpolarized MRI may be used to evaluate the effects of oncologic therapy on cardiac metabolism. Lastly, artificial intelligence and big data of imaging modalities may help predict and detect early signs of cardiotoxicity and response to cardioprotective medications as well as provide insights on the added value of molecular imaging and correlations with cardiovascular outcomes. In this review, the current imaging modalities used to assess for cardiotoxicity from cancer treatments are discussed, in addition to ongoing research on targeted molecular radiotracers, hyperpolarized MRI, as well as the role of artificial intelligence (AI) and big data in imaging that would help improve the detection and prognostication of cancer-treatment cardiotoxicity.

## Introduction

Cancer incidence is expected to increase by 50% by 2050, but over the past two decades, cancer mortality has improved in part due to earlier detection *via* screening and the advent of novel therapies such as tyrosine kinase inhibitors (TKI) for cancers like chronic myelogenous leukemia (CML), liver, gastrointestinal and lung cancers, as well as immunotherapy, such as checkpoint inhibitors, for metastatic disease and an expanding list of indications including triple negative breast cancer, lung cancer, melanoma, bladder cancer, and renal cell cancer (1–6).

However, with the rise of newer oncologic therapies, there have been a spectrum of adverse cardiovascular toxicities including cardiomyopathy (CM), myocardial infarction, myocarditis, arrhythmia, hypertension (HTN) and thrombosis that have been associated with these agents. More traditional cardiotoxic agents like anthracyclines (i.e., doxorubicin), one of the most widely used class of chemotherapeutics due to improved overall cancer and survival outcomes has been shown to alter myocardial energetics, promote mitochondrial dysfunction, increase reactive oxygen species levels leading to activation of matrix metalloproteases, inhibit topoisomerase IIb and cause DNA strand breaks, thereby promoting cardiomyopathy (7–9).

HER2 inhibitors like trastuzumab has also been shown to increase risk of CM *via* antagonizing important pro survival as well as other important signal transduction pathways for metabolism in the heart (10). Platinum agents like cisplatin have been shown to increase oxidative stress and increased apoptosis and has been associated with cardiomyopathy in rare instances (11). Alkylating agents like cyclophosphamide, which can cause oxidative damage and direct endothelial cell damage have been linked to myocarditis and cardiomyopathy (12). Antimetabolites like 5 fluorouracil (5FU), which is commonly used in head and neck cancers as well as gastrointestinal cancers has been shown to increase risk of coronary vasospasm and myocardial infarction (13, 14). Multiple myeloma therapies (bortezomib, lenalidomide) and vascular endothelial growth factor (VEGF) inhibitors like bevacizumab have been associated with thrombosis and hypertension by promoting endothelial cell dysfunction (15–18). TKIs like ibrutinib has been associated with atrial fibrillation, while other TKIs such as ponatinib, sorafenib, sunitinib have been associated with CM and myocardial infarction (MI) (19–21).

Of the close to 2 million patients diagnosed with cancer in 2019, it is estimated that 38.5% are eligible for ICI therapy (22, 23). In addition to increased risk of myocarditis, pericarditis and vasculitis, immune checkpoint inhibitors (ICI) have been associated with increased risk of plaque rupture/acceleration of atherosclerosis and thrombosis (24). ICI myocarditis is characterized by lymphocytic infiltration with CD4 and CD8 cells and mortality is high if not identified and if left untreated (25).

**Figure 1:**
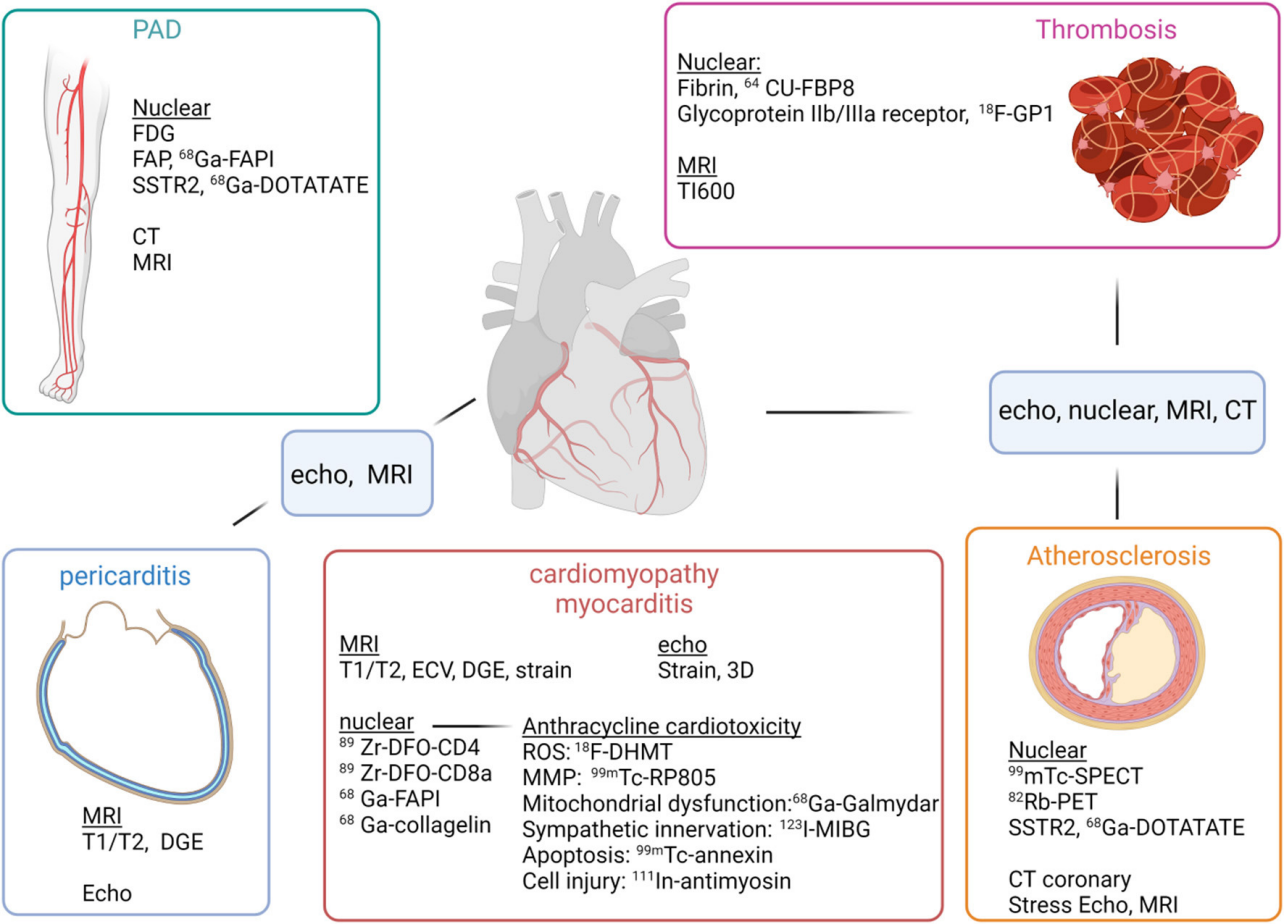
Imaging modalities and evaluation of cardiotoxicities of oncologic therapies. For evaluation of peripheral artery disease (PAD) (top left), FDG, FAP and SSTR2 imaging may be able to identify vulnerable plaque, while CT and MRI can help evaluate degree of stenosis. For evaluation of thrombosis (top right), nuclear imaging may be able to identify early clot formation with radiotracers directed at fibrin or glycoprotine IIb/IIIa, and MRI can use a long inversion time to identify thrombus, as with TI600. For evaluation of cardiomyopathy/myocarditis (middle), echo and MRI can evaluate ejection fraction as well as myocardial strain. For myocarditis, MRI can evaluate tissue characteristics such as T1, T2 and DGE, which are now components of the Lake Louise criteria for myocarditis. Nuclear can evaluate for T cell infiltration using tracers targeting CD4, CD8 cells. Tracers directed against FAP, such as ^68^Ga-FAPI has been shown to be increased in an animal model of checkpoint inhibitor myocarditis. Evaluation of pericarditis (bottom left), a complication of checkpoint inhibitors can be assessed by echo for detection of pericardial effusion, but with greater specificity MRI can identify edema and DGE. Atherosclerosis (bottom right) can be evaluated by traditional SPECT and PET techniques to evaluate for perfusion with stress and rest. CT coronary is now first line for evaluation of those with intermediate risk chest pain to rule out obstructive disease. Stress MRI or DGE can also be performed to evaluate for prior myocardial infarction as well as myocardial viability.

Newer immunotherapies may also increase risk of myocarditis, such as cellular therapies like CART and molecular inhibitors such as CCR4 antagonist, mogamulizumab, which is used to treat T cell lymphomas (26–28). However, evaluation of the earliest signs of immune cell infiltration in the myocarditis process is limited (Table 1; Figure 1). Imaging modalities like echocardiography (echo) and magnetic resonance imaging (MRI) are routinely used to monitor and evaluate for the aforementioned oncologic therapy related cardiotoxicity, with both allowing for assessment of function and wall motion abnormalities and MRI allowing for additional tissue characterization using T1, T2, extracellular volume (ECV) and delayed gadolinium enhancement (DGE) assessment. While nuclear studies like multi-gated acquisition (MUGA) scans have fallen out of favor for the evaluation of cardiomyopathy mediated by oncologic therapy due to the higher sensitivity, and availability of echo and MRI, emerging nuclear imaging using molecularly targeted radiotracers may confer more specificity and help elucidate the mechanisms of cardiotoxicity, many of which are already in clinical use for oncology purposes and thus can be adapted to evaluate their signal/role in cardiotoxicity (Table 1). In addition to molecular targets, hyperpolarized MRI has emerged as a potential imaging modality to evaluate effects of oncologic therapy on cardiac metabolism and has reached human studies. Finally, artificial intelligence and big data of imaging modalities including electrocardiograms may be able to help predict and detect early signs of cardiotoxicity and response to cardioprotective medications once cardiomyopathy develops but also help provide insights on diagnostic and prognostic value of molecular based imaging. We review current imaging modalities used to assess for cardiovascular toxicities associated with oncologic therapies and highlight ongoing research in the areas of molecular imaging, targeted molecular radiotracers and hyperpolarized MRI as well as the role of artificial intelligence (AI) and big data in imaging that would help improve detection, prognostication of oncologic therapy related cardiotoxicity.

**Table 1:** Cancer therapy, associated CV toxicity and imaging assessment.

**Cancer therapy**	**Associated CV toxicity**	**Imaging modality/method for evaluating cardiotoxicity**	**Novel molecular imaging approaches**	**Stage preclinical vs. clinical**
**Anthracyclines**: Doxorubicin, daunorubicin	Cardiomyopathy (29) Early stages of toxicity	MRI, echo, nuclear	Molecular nuclear imaging for cardiotoxicity:	
			SPECT radiotracers:	
			^123^I-meta-iodobenzylguanidine (MIBG) (30)	Clinical
			^99m^Tc-RP805 (31)	Preclinical
			^111^In-antimyosin (30)	Clinical (32)
			^99m^Tc-annexin (33)	Clinical (34)
			PET radiotracers:	
			^18^F-DHMT (35)	Preclinical
			^68^Ga-Galmydar (36)	Preclinical
			Changes in metabolism:	
			Hyperpolarized magnetic resonance (37)	Clinical
			^13^C pyruvate (38, 39)	
**Other:** Topoisomerase I/II inhibitors, taxols, cyclophosphamide, paclitaxel			Hyperpolarized magnetic resonance (37)	Clinical
**Platinum agents:** cisplatin, oxaliplatin, carboplatin				
**Checkpoint inhibitors**				
Pembrolizumab	Myocarditis (40), vasculitis, pericarditis (41, 42), atherosclerosis (43)	Echo for function/strain, MRI for function, tissue characterization i.e.,	Molecular imaging for myocarditis:	
Ipilimumab		MRI:	^89^Zr-DFO-CD4 and ^89^Zr-DFO-CD8a (44)	Clinical
Nivolumab		Edema/scar imaging	^68^Ga-FAPI (45)	Clinical
Atezolizumab		PET:		
Avelumab		^18^FDG to evaluate for vasculitis.	Fibrosis imaging:	
Cemiplimab		^82^Rb to evaluate for ischemic disease	^68^Ga-collagelin (46)	Preclinical
		SPECT:		
		^99m^Tc-tetrofosmin or ^99m^Tc-sestamibi to evaluate for ischemic disease		
**TKIs**				
Imatinib	HF (47)	MRI, echo, nuclear SPECT		
Bosutinib	Thrombosis (48)		Thrombosis imaging	
			Evaluation of fibrin	
			^64^CU-FBP8 (49)	Clinical trials (50)
			Evaluation of glycoprotein IIb/IIIa receptor	
Dasatinib	Thrombosis (51), HTN, QT prolongation (52)		^18^F-GP1 (53)	Clinical trial (53)
Ponatinib	Thrombosis (54), HF (55), HTN, ischemia	MRI, echo		
Nilotinib	Thrombosis, QTC prolongation (52)			
Ibrutinib	A Fib (19)			
Sunitinib	HF (56), HTN, QTC prolongation (57)	MRI, echo		
Sorafenib	MI, HF, HTN, QTC prolongation	CT coronary, PET/SPECT for ischemic evaluation	Hyperpolarized magnetic resonance	Clinical
			^68^Ga-DOTATATE (58)	Clinical (59)
Vendetanib	HF, HTN (60), QTC prolongation, TdP (61)			
Afatinib	None so far (62)			
Erlotinib	MI (rare) (63)			
Lapatinib	HF, QT prolongation (64)	MRI, echo		
Gefitinib	HF (65)	MRI, echo		
axitinib	HF, HTN (66)	MRI, echo		
bevacizumab	HTN, thrombosis		Hyperpolarized magnetic resonance to evaluate hypertensive stress (67)	Clinical
Trastuzumab	Heart failure (68–70)	MRI, ECHO, nuclear (MUGA)		
Pertuzumab				
Neratinib				
Tucatinib				
**Anti metabolite**				
5 FU	Coronary vasospasm (14, 71)	CT coronary, PET or SPECT to rule out obstructive disease	Hyperpolarized magnetic resonance	Clinical

## Current Imaging Modalities Used to Interrogate Oncologic Therapy Cardiotoxicity

### Echo and MRI in Evaluation of Cardiotoxicity

Cardiotoxicity due to anthracycline use (often dose dependent, but can occur at any dose) are common, up to 5% with cumulative doses <400 mg/kg, but up to 20% for those treated with 700 mg/kg or more (72). HER2 inhibitor mediated cardiomyopathy can occur in 5–10% of patients and is increased when given in conjunction with anthracyclines up to 27% (73, 74). Oncologic therapy mediated cardiomyopathy can be evaluated by traditional imaging modalities such as echo and MRI, which are able to evaluate wall motion, left and right ventricular function and even early signs of toxicity *via* changes in strain, namely global longitudinal strain (75, 76). The European Society for Medical Oncology (ESMO) and the American Society of Echo (ASE) recommend 2D/3D echo or MRI for assessing left ventricular function including strain for monitoring of known cardiotoxic therapies such as anthracyclines or anti-Her2 therapies and the American Society of Clinical Oncology (ASCO) recommends echo or MRI as first line imaging modalities with MUGA as a second line if echo/MRI are not available or if not technically feasible for MRI (77–81). Due to reduced variability compared to 2D echo, 3D echo or MRI are recommended for sequential follow up (82).

In addition to being the gold standard for volumetrics and ejection fraction, MRI has additional evaluation capabilities including tissue characterization for injured cells such as changes in ECV and increased native T1 times, shown with anthracycline use and increased T2 relaxation times with anthracycline toxicity (83–86). The presence of DGE post trastuzumab, a HER2 inhibitor, was associated with cardiomyopathy (87).

### Strain as a Predictor of Cardiomyopathy

Feature tracking global longitudinal strain (GLS) was first used in echo to show that it could be predictive of future cardiomyopathy in multiple studies of cancer patients undergoing cardiotoxic chemotherapy with anthracycline or trastuzumab. For example, an increase in GLS >12 or 15% was associated with a significant drop in LVEF >10% 6 months after in several studies (88, 89). MRI has subsequently shown that use of tagging, feature tracking strain or fast strain encoded (SENC) assessment are sensitive and highly accurate in detecting subclinical cardiotoxicity as evidenced by an increase in GLS for patients on cardiotoxic chemotherapy such as anthracyclines, with SENC having a higher accuracy that was less dependent on loading conditions (90–94). However, strain assessment in MRI is largely used in a research setting and is not routinely used in the clinical practice yet.

### MRI Evaluation of Adverse Immune Related Cardiac Events

ICI myocarditis can occur in 1–2% of patients and has a high mortality of up to 50% if untreated (25, 95). MRI has become a work horse for evaluation of immunotherapy related cardiotoxicities. In addition to T1, and ECV changes, T2 abnormalities allow for assessment of myocardial edema in patients on checkpoint inhibitors with concern for myocarditis or pericarditis and DGE, a marker of myocardial injury or scarring is another tissue characterization parameter that can evaluate for immunotherapy toxicities. MRI is recommended by specialty society guidelines as part of the evaluation and monitoring of ICI myocarditis using the Lake Louise criteria, updated in 2018 to require both increased myocardial signal intensity ratio >2 or increased myocardial relaxation times or visible myocardial edema in T2-weighted images and increased myocardial relaxation times or extracellular volume fraction or DGE in T1-weighted images for the imaging diagnosis of myocarditis (80, 96–100). However, DGE is non-specific and cannot distinguish from cell damage vs. end stage fibrosis and current standard clinical imaging modalities are lacking in assessment of potential molecular correlates, such as collagen deposition and scar. Thus, molecularly targeted imaging tracers may shed light on both mechanism and help increase the specificity of cardiac imaging findings.

## Molecular Targeted Nuclear Imaging Modalities to Evaluate Oncologic Therapy Related Adverse Cardiovascular Pathologies

### Molecular Nuclear Imaging for Evaluation of Anthracycline Cardiotoxicity

Anthracycline mediated cardiotoxicity has been associated with an increase in reactive oxygen species (ROS) levels in the heart. ROS levels have been shown to confer cardiotoxicity by increased apoptosis, inflammation, mitochondrial dysfunction and activation of matrix metalloproteases (31). Molecular nuclear imaging studies have helped shed light on mechanisms of anthracycline mediated cardiotoxicity. Increased ROS levels in an animal model of doxorubicin cardiotoxicity showed that a novel PET tracer, ^18^F-labeled radioanalog of dihydroethidium, [^18^F]-6-(4-((1-(2-fluoroethyl)-1H-1,2,3-triazol-4-yl)methoxy)phenyl)-5-methyl-5,6 dihydrophenanthridine-3, 8-diamine ([^18^F]·DHMT), which targets superoxide, was able to reveal an elevation in superoxide levels in the heart at least 2 weeks prior to a drop in the left ventricular ejection fraction (35). ROS activation of MMPs downstream can then promote adverse cardiac remodeling (101). Renin-angiotensin-aldosterone system (RAAS) activation has been shown to augment the progression of anthracycline induced cardiotoxicity and inhibition *via* RAAS inhibitors like angiotensin receptor blockers or angiotensin converting enzyme inhibitors have been able to prevent and treat anthracycline mediated cardiomyopathy (102, 103). Use of a novel angiotensin receptor-neprilysin Inhibitor, sacubitril/valsartan in a rodent model of anthracycline cardiotoxicity was able to attenuate cardiotoxicity. MMP imaging of activated MMPs using SPECT radiotracer ^99m^Tc-RP805 showed that sacubitril/valsartan in conjunction with doxorubicin was able to significantly attenuate MMP activation as well as prevent a decline in LVEF compared to doxorubicin alone vs. doxorubicin and valsartan groups. Myocardial MMP activity as assessed by ^99m^Tc-RP805 uptake was inversely related to left ventricular ejection fraction (31). In addition to MMP activation and adverse remodeling, ROS can also injure endothelial cells. Anthracycline use has been associated with capillary loss in the heart in some rodent models and protection of endothelial cells with vascular endothelial growth factor-B (VEGF-B) treatment led to preservation of capillary mass (104).

ROS has also been shown to confer mitochondrial dysfunction. Disruption of mitochondrial membrane potential in mitochondrial dysfunction mediated by anthracycline can be evaluated by ^68^Ga-Galmydar. In a rodent model, uptake of ^68^Ga-Galmydar was reduced by 2-fold with anthracycline treatment compared to control and in H9c2 rat cardiomyoblasts, this was associated with activation of the apoptosis cascade (36).

**Figure 2:**
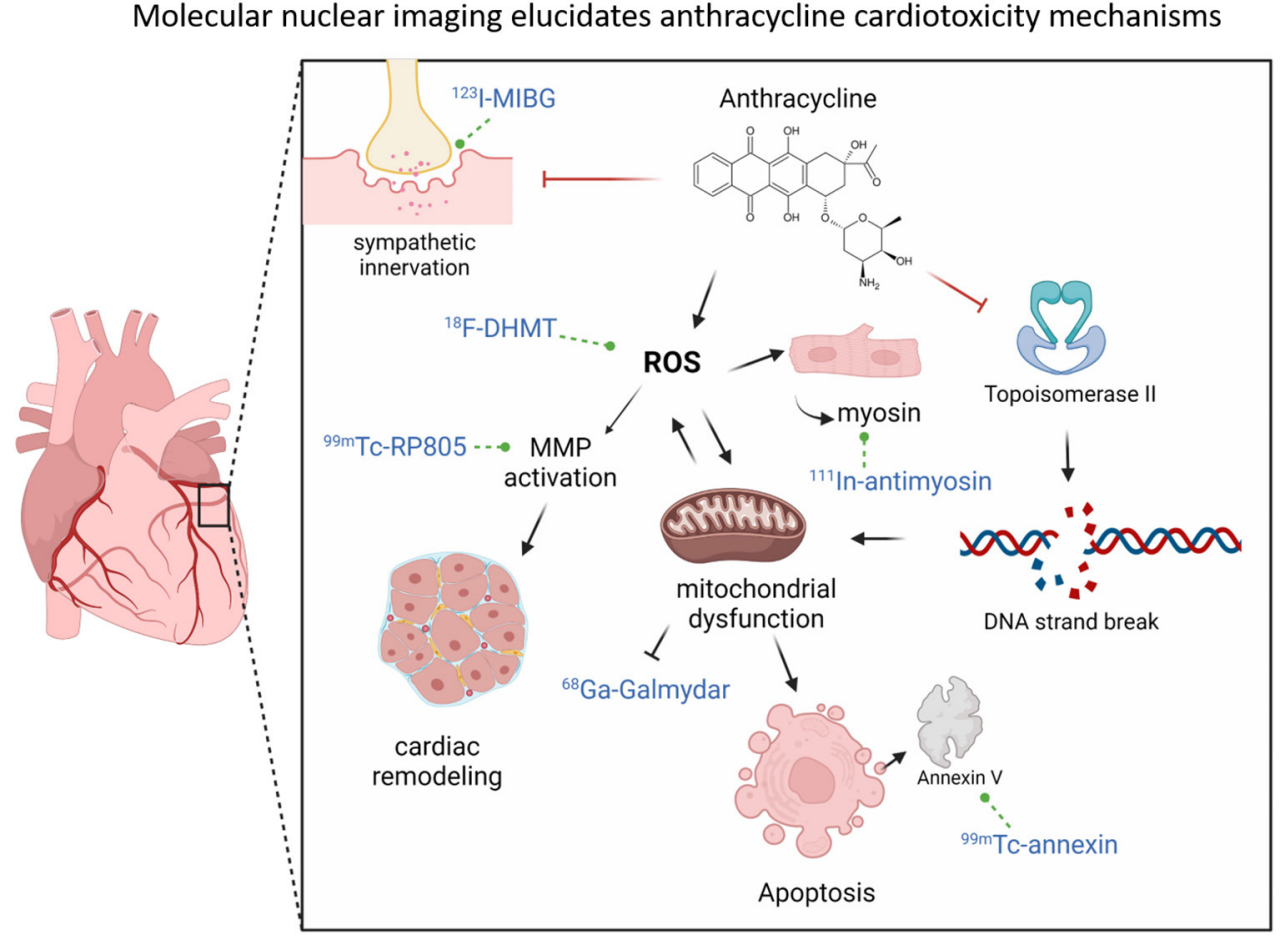
Molecular nuclear imaging elucidates anthracycline cardiotoxicity mechanisms. Anthracyclines can increase ROS levels (which can be assessed by nuclear tracer ^18^F-DHMT), which can activate MMPs (which can be assessed by ^99m^Tc-RP805) (bottom left), leading to adverse cardiac remodeling. ROS levels can also promote mitochondrial dysfunction, which can disrupt the mitochondrial membrane potential and thereby reduce ^68^Ga-Galmydar uptake (middle bottom). Mitochondrial damage can lead to apoptosis, which can be detected by Annexin V positivity (detected by ^99m^Tc-Annexin (bottom right). Damage to cardiomyocytes can lead to release of intracellular myosin, which can thereby be assessed by (105). In-myosin (right of ROS). In addition to ROS increase, anthracyclines can also directly bind and inhibit Topoisomerase II, which can lead to double-stranded DNA breaks (right) and cause further mitochondrial dysfunction and prevent mitochondrial regeneration. Finally, anthracyclines can lead to impaired sympathetic innervation over time for mechanisms that are unclear but is associated with cardiac dysfunction and this can be assessed by ^123^I-MIBG uptake (top left).

Early markers of anthracycline cardiotoxicity include an increased uptake of indium-111-labeled antimyosin in the heart, which occurs due to myocyte damage and subsequent association of antimyosin with myosin, which is normally intracellular. Increased uptake of ^111^In-antimyosin in patients on anthracycline was associated with LV dysfunction (30). Detection of the earliest stages of apoptosis can also signal early toxicity. Annexin V has a high affinity for phosphatidylserine, which gets exposed on the cell surface during apoptosis. Use of annexin V imaging has allowed for detection of cells undergoing apoptosis. In a rodent model of doxorubicin cardiotoxicity, radiolabeled annexin V, ^99m^Tc-annexin was used to visualize apoptosis that corresponded to histological evidence of apoptosis on TUNEL staining (33). Finally, sympathetic nervous innervation of the myocardium has also been shown to be disrupted with anthracycline toxicity. An assessment of myocardial sympathetic innervation impairment was done by evaluating a radiotracer that is an analog of norepinephrine, iodine-123-labeled meta-iodobenzylguanidine (^123^I-MIBG). A decrease in ^123^I-MIBG uptake with increasing cumulative doses of anthracyclines in human patients was associated with LV dysfunction. However, it takes higher cumulative doses of anthracycline to see a drop in ^123^I-MIBG uptake, thus this agent would be less useful if earlier detection of toxicity is desired. However, ^123^I-MIBG is clinically available and routinely used to evaluate for adrenaline secreting tumors (30) (Figure 2).

### CD4, CD8 Imaging in ICI Myocarditis

Molecularly targeted radiotracers in nuclear medicine are emerging to evaluate processes such as fibrosis, inflammation and thrombosis, extending beyond nuclear cardiology's traditional use to evaluate perfusion deficits in ischemic heart disease *via* single photon emission computed tomography (SPECT) and positron emission tomography (PET), tissue viability or inflammation with PET fluorodeoxyglucose (FDG), which evaluates for glucose uptake predominantly by inflammatory cells, such as myeloid and T cells (106). These processes are common adverse effects of oncologic and immunotherapies.

Detection of the earliest signs of myocardial inflammation in ICI myocarditis, which occurs in 1-2% of patients on these agents remains a clinical challenge (95, 107). The ability to detect the initial infiltration of inflammatory cells such as CD4 or CD8 cells before injury has occurred could help reduce morbidity and high mortality associated with this condition (25). Emerging molecularly targeted probes against CD4, ^89^Zr-DFO-CD4 and CD8 cells, ^89^Zr-DFO-CD8a may be a potential avenue to detect inflammation at these earliest of stages, which can prompt more frequent follow ups, biomarker checking and earlier therapy (44). Determining specificity of these findings will also be important as to avoid withholding cancer fighting immunotherapy or treatment with steroids, which may potentially lower the efficacy of the immunotherapy agent (108–110). Checkpoint inhibitors have been shown to accelerate atherosclerosis and increase risk of plaque rupture in addition to the risk for myocarditis and pericarditis by driving increased inflammatory cells, including CD8 T cell infiltration into plaques in animal models and patients on checkpoint inhibitors (43, 111, 112). Thus, evaluation of atherosclerotic lesions with CD8 radiotracers, may be able to identify those at risk for myocardial infarction in patients on checkpoint inhibitor therapy.

### Detection of Vulnerable Plaque

Both checkpoint inhibitor use and certain TKIs like ponatinib and sorafenib have been associated with increased risk of myocardial infarction (43, 113). ICIs have also been associated with increased risk of stroke (114). Use of ICIs have been associated with increased infiltration of CD3, CD8 and CD68 cells, markers for T cells and macrophages respectively into atherosclerotic lesions (115). Increased somatostatin receptor 2 (SSTR2) on the cell surface of inflammatory macrophages is a marker of macrophage activation. In a study of symptomatic stroke patients, increased uptake of SSTR2 in culprit vessels assessed by PET tracer ^68^Ga-DOTATATE was shown to predict plaque rupture (58). Thus, evaluation of SSRT2 levels in patients on ICI therapy may help identify vulnerable plaques and warrants further investigation. The mechanisms for TKI mediated MI on the other hand are attributed to endothelial cell dysfunction and activation of apoptosis pathways, although direct evidence for MI mechanisms are still lacking, thus further research would be needed to see if macrophage activation is involved and whether activated macrophage imaging would help risk stratify patients on these TKIs (113).

### FAP Imaging in ICI Myocarditis

Another potential marker of early stages of ICI myocarditis is fibroblast activating protein (FAP), which is a protein that gets significantly upregulated in cancer tissue, atherosclerosis, arthritis and fibrosis. It is emerging as an imaging marker for fibroblast activation and fibrosis (116, 117). A PET radiotracer tracer targeting FAP is ^68^Ga-FAPI. In a recent study, ^68^Ga-FAPI was shown to be a potential early marker of ICI myocarditis with median standardized uptake values (SUV) 1.79 (IQR 1.62, 1.85) in myocarditis patients vs. 1.15 (IQR 0.955, 1.52) in non-myocarditis patients (45). FAP has also been used to evaluate post myocardial infarction fibrosis, but its level in the blood vessels and myocardium of patients on checkpoint inhibitors is unclear (118, 119).

### PD1 Imaging as a Potential Risk Factor for ICI Myocarditis

Another challenge with checkpoint inhibitor myocarditis is trying to figure out who is at increased risk. Programmed cell death protein 1 (PD1), a target of checkpoint inhibitors like pembrolizumab and its expression on cardiomyocytes warrants additional research as a potential risk factor. PET radiotracer, ^64^Cu-DOTA-pembrolizumab can detect PD1 in rodent hearts as well as on the surface of human blood cells and may be used in such an investigation (120).

### MRI DGE Limitations in Fibrosis Assessment and Collagen Imaging

**Figure 3:**
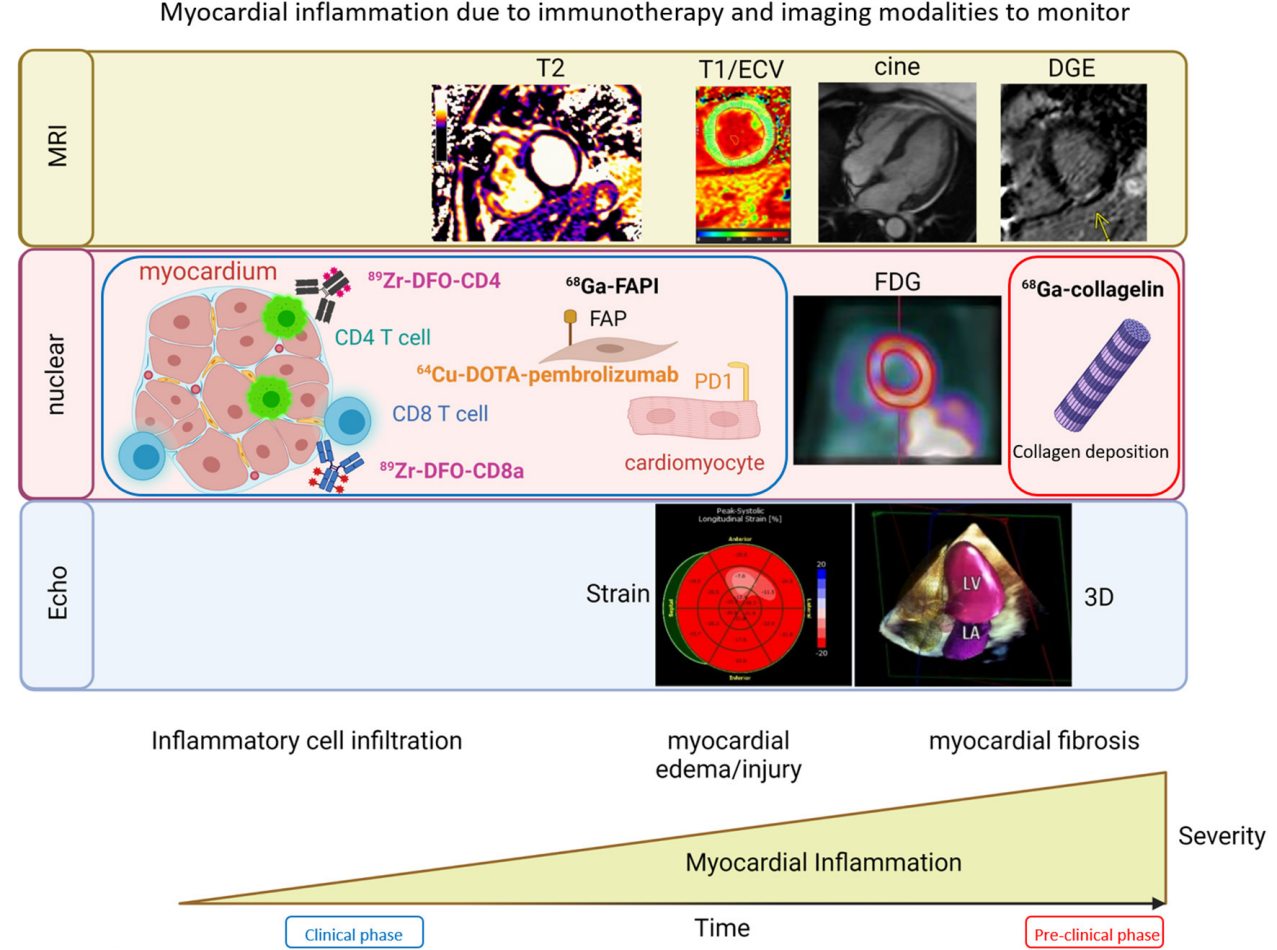
Imaging modalities in the evaluation of immunotherapy related cardiotoxicities. Imaging modalities that can be used to monitor myocardial inflammation due to immunotherapy include: **MRI** (top) using tissue characterization assessments such as T2, T1/ECV, delayed gadolinium enhancement (DGE) and cine to evaluate wall motion and function; **Nuclear Imaging** (middle) approaches involving molecularly targeted probes conjugated to radiotracers facilitating evaluation of CD4 cells with ^89^Zr-DFO-CD4, CD8 cells with ^89^Zr-DFO-CD8, early signs of fibrosis with fibroblast activation protein (FAP), expression of PD1 on cardiomyocytes, which can be seen with ^64^Cu-DOTA-pembrolizumab and may reflect increased risk of checkpoint inhibitor myocarditis, FDG that allows for monitoring of inflammation, and the final stages of inflammation with tissue damage and fibrosis and scar deposition assessed with collagen imaging with ^68^Ga collagelin; **Echocardiography** (bottom) is able to evaluate regional and global strain to detect signs of chemotherapy related toxicity and myocarditis.

A higher burden of DGE and presumed scarring in hypertrophic cardiomyopathy is associated with worse cardiovascular and death outcomes (121, 122). In a retrospective study of ICI myocarditis patients who underwent cMRI, DGE evaluation did not correlate with cardiovascular outcomes, nor fibrosis, with only 35% of pathology proven fibrosis cases showing DGE on MRI (96, 121, 123, 124). Further, of the 56 patients with histopathology available either through biopsy or autopsy, 98% had lymphocytic infiltration but only 38% had DGE and 26% with T2 positivity (96). Thus in addition to evaluation of lymphocytic infiltration with targeted radiotracers for CD4 and CD8 cells to identify early stages of myocarditis and increase sensitivity of diagnosis, late stages of myocardial injury that can result in scar and thus collagen deposition can be evaluated by radiotracers targeting collagen. The PET radiotracer ^68^Ga-collagelin targets collagen, which can help quantify the burden of scarring or end stage fibrosis, which was shown to be able to detect pulmonary fibrosis in a mouse model of bleomycin induced pulmonary fibrosis and correlated with fibrosis on pathology (46) (Figure 3). MRI with DGE is able to evaluate for possible scarring, but it is not able to distinguish between early vs. late stage fibrosis, with the former having potential reversibility and may partially explain the differential outcomes we see between HCM and ICI myocarditis patients when it comes to the differences in the fibrosis processes between the two conditions and correlation of scar burden as quantified by DGE and outcomes (125). There is also a MRI collagen type I targeted probe EP-3533 that is conjugated to gadolinium, which was shown to be able to visualize pulmonary, liver and bowel fibrosis in rodent models, but these have not yet advanced to use in humans (126–128).

### Thrombosis Imaging

Pathologic thromboses like pulmonary embolism (PE), deep vein thrombosis (DVT) carries high morbidity and mortality (129). Cancer patients are at increased risk of thrombosis and some of their oncologic therapies can increase that risk further (130, 131). ICI, VEGF inhibitors and lenalidomide have been associated with increased thrombosis risk. Increasing the sensitivity of diagnosing blood clots so treatment can be timely instigated may help avoid complications and help improve outcomes (132–134). Radiotracers that can target fibrin, a molecular precursor of blood clotting can be useful in detection of blood clots. PET radiotracer ^64^CU-FBP8 can target fibrin and has been used to identify thrombi in animal models, particularly earlier stages of clots (49). Another PET radiotracer, ^18^F-GP1 that targets the glycoprotein IIb/IIIa receptors on activated platelets and has been demonstrated to detect venous thrombosis and arterial thromboses (53, 135). A phase 1, first-in-human study of ^18^F-GP1 positron emission tomography for imaging acute arterial thrombosis is underway (53). These PET thrombosis imaging agents may be of utility for detection of DVTs and PEs in cancer patients, especially for those who may have contraindications to contrast, such as those with chronic kidney disease or those who have an allergy to contrast.

## Molecular MRI and MR Spectroscopy

### Hyperpolarized MRI for Evaluation of Cardiac Metabolism *in vivo*

As the human heart failures, it has been shown to shift its metabolism from predominantly fatty acid oxidation to more glucose utilization (136). Changes in oxidative phosphorylation or substrate utilization may reflect early signs of cardiotoxicity, yet *in vivo* real time detection of cardiac metabolism has been limited to small studies with radioactive tracers using PET. More recently, substrate utilization and metabolism have been evaluated using magnetic resonance (MR) imaging and spectroscopy. Hyperpolarized carbon-13 (^13^C) labeled pyruvate imaging is different from standard clinical MRI using gadolinium contrast, in that it provides information on how tissue uses carbon-based nutrients (37). In rodent models of anthracycline cardiotoxicity, carbon-13 MR spectroscopy (MRS) was used to assess changes to oxidative phosphorylation and tricarboxylic acid (TCA) cycle flux *in vivo*. These studies showed that doxorubicin lead to reduced cardiac oxidative phosphorylation in a rat model as evidenced by increased ^13^C lactate production (38). First in human MRS was used to evaluate tumor metabolism in prostate cancer and ongoing clinical trials are evaluating hyperpolarized MR in tumor metabolism and correlations with outcomes in prostate and pancreatic cancer (137–139). First use of hyperpolarized ^13^C metabolic MRI in human heart involved evaluation of pyruvate metabolism in healthy individuals (39). Hyperpolarized MR imaging may allow for visualization of changes in cardiac energetics, particularly from fatty acid metabolism to more glucose utilization in an evolving cardiomyopathy in response to cardiotoxic chemotherapy and to evaluate response to cardioprotective medications such as beta blockers and angiotensin converting enzyme inhibitors in real time (140).

### Apoptosis Evaluation by MRI

Various chemotherapy agents, most notably anthracyclines are known to increase cardiomyocyte apoptosis. Molecular MRI probes conjugated to superparamagnetic iron oxide (SPIO) and human annexin was shown to be able to visualize apoptosis in real time in a rodent model following ischemia and post doxorubicin exposure, but these MRI molecular probes have not gone beyond animal studies thus far but have the potential to detect early signs of cell death in the myocardium (105, 141).

### Inflammation Imaging by MRI

In addition to T1, ECV and T2 signal changes, use of ultrasmall superparamagnetic particles of iron oxide (USPIOs) in MRI may confer insights on inflammation *via* increased macrophage activity. USPIOs have been shown to be taken up by macrophages and correlates with plaque inflammation in animal studies (142). In a study of patients with severe carotid stenosis, uptake of USPIOs corresponded to inflamed plaques on histology. Uptake of USPIOs induced areas of signal loss on ${\text{T}}_{2}^{*}$-weighted magnetic resonance imaging within the vessel wall. Whether this can help predict plaque vulnerability in those on checkpoint inhibitors or help identify ICI myocarditis is untested and warrants further investigation (143). However, this has been used clinically and may have potential to distinguish vulnerable plaque from less vulnerable plaque.

### Barriers to Advancing Molecular Imaging

For the molecular imaging tracers that are already clinically used, barriers to use include radiation exposure, so deciding who should get the test, when to get it and how often will have to be established. For example, if FAP is associated with ICI myocarditis as a potential early marker, then perhaps it should be obtained when there is suspicion for myocarditis or when troponin becomes positive. Timed with evaluation of this marker for residual disease, it can also help with monitoring of resolution of myocarditis, potentially complementing cardiac MRI or taking place of MRI for those who cannot tolerate MRI, which is usually used for monitoring. Access is another challenge. Access to molecular nuclear studies are often available through large hospital systems and for agents with shorter radioisotope half-lives like Gallium-68 (^68^Ga) with average half-life of 68 min, an onsite germanium-68/gallium-68 generator is needed along with accompanying nuclear accreditation, thus, more rural hospitals or private practices may have to refer out to larger centers in order to obtain these tests at high volume imaging centers (144). Finally, nuclear studies tend to be more expensive than echo and either on par or more expensive than MRI studies due to the costs associated with radiolabeled probes, thus being able to get these studies approved can also be a challenge for providers even if it is clinically used and indicated. For the molecular tracers that are in the preclinical stage, the usual barriers exist for clinical translation, including establishing safety, a favorable target to noise ratio in humans and correlation with outcomes to achieve FDA approval and ultimately clinical use. For those radiotracers that are already in clinical use for oncology indications, such as FAP, CD4, CD8 and PD1, incidental detection in the heart and correlation with outcomes is possible and can be further explored for future dedicated cardiac imaging and may provide unique clinical value. The power of machine learning, artificial intelligence and big data in evaluation of imaging signals can help unlock patterns that humans may not readily be able to see, such as in a recent evaluation of cardiac fibrosis by T1 imaging by MRI and be able to correlate these imaging findings with outcomes (145).

## Role of Artificial Intelligence (AI) and Big Data in Cardio-Oncology and Imaging

### Overview of Current AI Applications in Cardio-Oncology

**Figure 4:**
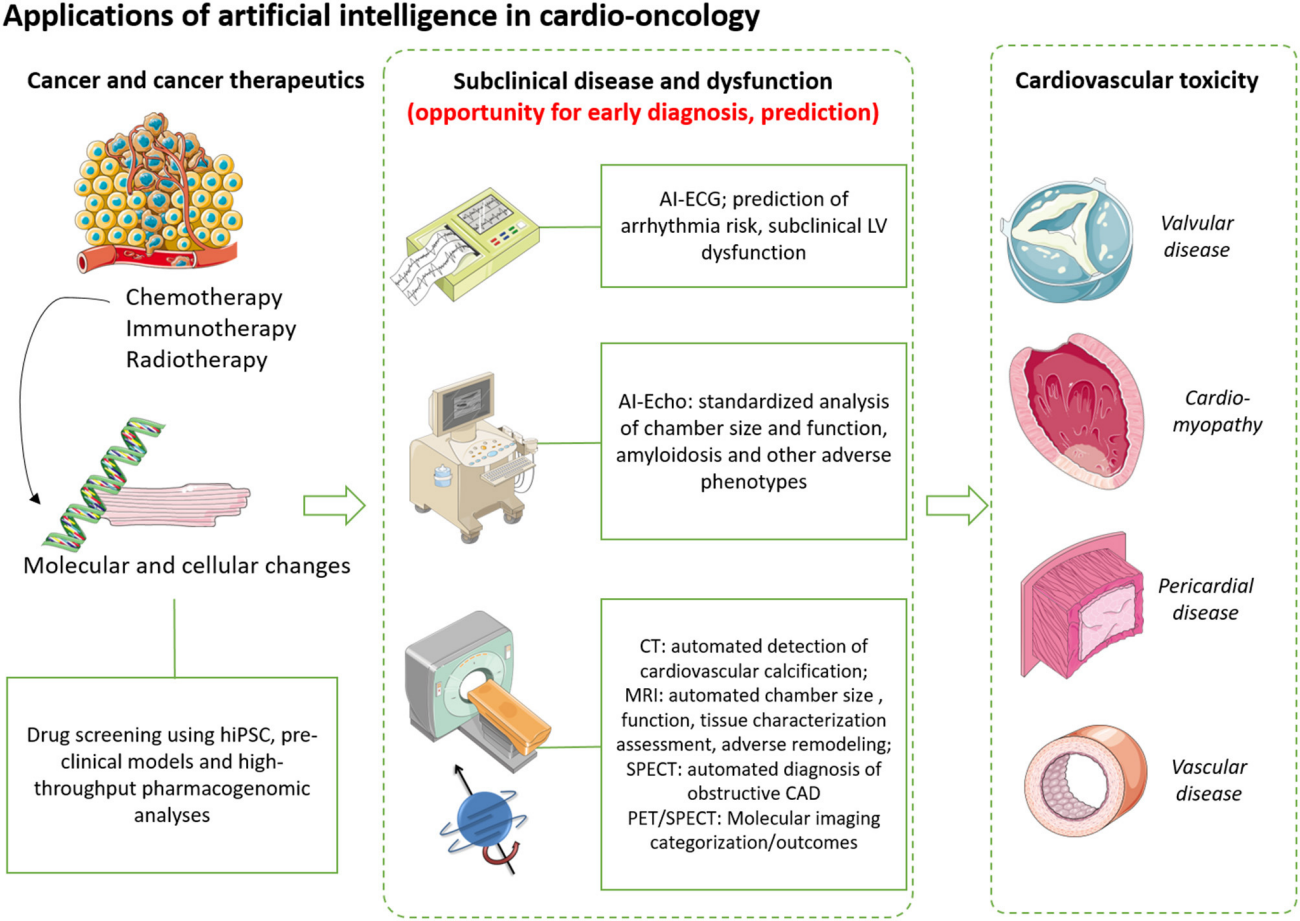
Applications of artificial intelligence, big data in cardio-oncology. Artificial intelligence (AI) can improve our understanding of the early molecular and phenotypic changes that occur prior to the development of clinical cancer therapeutics-related cardiac dysfunction. Machine learning approaches enable high-throughput screening of novel therapeutics using preclinical models, such as induced pluripotent stem cells as well as *in silico* simulations using libraries of drugs and molecular targets. In the clinical setting, AI can improve risk prediction of left ventricular dysfunction, arrhythmias as well as facilitate accurate and standardized assessment of chamber size, function and coronary calcification, all hallmarks of cardiovascular disease that can be caused or exacerbated by cancer therapeutics. Therefore, AI offers an opportunity for early diagnosis and deployment of strategies to prevent the progression to overt cardiovascular disease. Images have been reproduced under a Creative Commons Attribution 3.0 Unported License from smart.servier.com. CAD, coronary artery disease; CT, computed tomography; ECG, electrocardiography; hiPSC, human induced pluripotent stem cell; LV, left ventricular; MRI, magnetic resonance imaging; SPECT, single photon emission computed tomography.

Artificial intelligence (AI), through the training of machine and deep learning models, has shown remarkable potential in the prevention and diagnosis of cancer therapeutics-related cardiac dysfunction (CTRCD). With applications across all stages of the natural history of CTRCD, AI can assist scientists and physicians in screening for molecular interactions between novel therapeutic agents and the cardiovascular system, as well as detecting subclinical cardiovascular effects prior to the development of overt clinical disease (Figure 4).

At the pre-clinical stage, AI techniques have been used for high-throughput screening of cancer agents using a variety of disease models. These range from human induced pluripotent stem cell-derived cardiomyocytes (hiPSC-CMs) exposed to antineoplastic agents, screening of drug libraries to detect agents that interact with channel proteins resulting in QT prolongation, all the way to exome sequencing to identify variants in cardiac injury pathway genes that protect against anthracycline-induced cardiotoxicity and dual transcriptomic and molecular machine learning to predict different types of cardiotoxic response (146–150). Such approaches can de-risk early-stage drug discovery but also contribute to post-marketing surveillance to maximize patient safety. On the same note, pharmacovigilance in cardio-oncology can be assisted by machine learning-guided monitoring of electronic health records that includes patient demographics, echocardiography, laboratory values to detect signals suggestive of increased cardiac risk with specific therapies or practices (151, 152).

For therapies that form the mainstay of cancer therapy, ranging from chemotherapy to immunotherapy and radiation therapy, active surveillance protocols have been proposed and implemented, particularly for therapies with known cardiotoxic effects, such as anthracyclines and HER-2/neu inhibitors. Here, non-invasive cardiac imaging (by means of transthoracic echocardiography and/or magnetic resonance imaging (MRI)) and electrocardiography (ECG) represent the modalities of choice in the screening of conditions, such as anthracycline-induced cardiotoxicity and immune checkpoint-induced myocarditis (78, 153). Whereas AI applications in cardiovascular imaging have traditionally been developed in the general population, shared phenotypes seen in both CTRCD and non-cancer-related cardiac dysfunction, may extend the use of these technologies to cardio-oncology.

An expanding body of research has in fact demonstrated the ability of deep learning-enhanced interpretation of ECG in screening for and improving the diagnosis of left ventricular dysfunction, essentially functioning as a gatekeeper to the use of more advanced imaging modalities (154). It is notable that this tool was tested in a randomized controlled trial and demonstrated effectiveness in increasing the early diagnosis of decreased left ventricular ejection fraction (LVEF) without an increase in the use of echocardiography (155). Similarly, AI-guided ECG assessment can also predict the future incidence of atrial fibrillation (156). In childhood cancer survivors, machine learning algorithms of baseline and follow up ECGs were able to predict future cardiomyopathy (157). However, whether these results generalize to cardio-oncology, such as in the monitoring of anthracycline or Herceptin mediated cardiotoxicity, or ibrutinib-associated atrial fibrillation remains unknown and should be explored in future studies (158, 159).

AI has contributed to a more efficient and standardized interpretation of several non-invasive cardiovascular imaging modalities. For instance, in the field of transthoracic echocardiography, deep learning video-based models now enables fast and automated calculation of LVEF, with variance that is comparable to that or even lower of a human observer (160, 161). Similarly, combined assessment of ECG- and echocardiography-derived AI models has shown good discrimination in detecting cardiac amyloidosis, a rare disorder that is however more prevalent among patients with cancer compared to the general population (162). Similar approaches can be found in the field of computed tomography (CT) imaging, where automated tools enable an accurate assessment of coronary artery calcium burden, which can be generalized to both gated and non-gated CT scans of the chest, with the latter often used in the staging or monitoring of patients (163, 164). Therefore, such tools may refine a patient's baseline cardiovascular risk and inform risk-benefit discussions about the deployment of potentially cardiotoxic therapies. Finally, automated chamber size quantification, tissue characterization parameters such as T1, T2, extracellular volume and functional indices that can be extracted from cardiac MRI images can have the ability to confer insights into cardiotoxicity including the potential to identify early to late cardiotoxicity mediated by chemotherapy or immunotherapy agents *via* detection of changes in chamber size, abnormal T1, T2 relaxation times and delayed gadolinium enhancement patterns (86, 95, 96, 99, 145, 165–167). Deep learning models have also shown promise in the standardized interpretation of functional nuclear modalities, such as SPECT (single photon emissions computed tomography) myocardial perfusion imaging with good discrimination for the presence of obstructive coronary artery disease (168). However, as these tools become clinically available, prospective validation and possibly recalibration specifically in patients with cancer will be required to ensure their validity and generalizability.

### Strengths and Weaknesses of Current Methods and Barriers for Clinical Translation

To better understand the strengths and weaknesses of AI applications in cardio-oncology, one first needs to review key definitions. AI refers to the ability of an automated system to perform tasks that are typically characteristic of human intelligence, such as image and pattern recognition, as well as prediction and classification. *Machine learning* describes the process by which a system gains the ability to perform such tasks. This learning process can be further divided into *supervised* and *unsupervised learning*. The former describes the analysis of labeled datasets with the goal of predicting the label of a given datapoint based on a set of independent predictors. The latter refers to the analysis of unlabeled and unclassified datasets where the algorithm attempts to discover patterns within the data on its own. Algorithms may range from traditional regression models to deep neural networks, consisting of multiple layers of neurons and nodes which operate in a manner similar to the human brain (169, 170). However, independent of the algorithm used, machine learning systems rely on high-quality input to deliver high-quality output. This is where “big data” become relevant, describing the need for datasets that are large enough to ensure adequate variance, remain representative of their original and target populations, enable time-efficient analyses and have been carefully rather opportunistically curated to address a specific question (171).

With those key concepts in mind, some of the limitations of machine learning applications in cardio-oncology become apparent. First, cardiovascular disease is often listed as an exclusion criterion in major cancer trials, thus resulting in under-representation of patients with cardiovascular disease in pivotal cancer trials (172). However, the inclusion of cardiovascular outcomes in cancer trials will be able to help fill this data gap if sufficient baseline and follow up data are acquired (molecular biomarkers, baseline imaging prior to oncologic therapy and follow up that can be used as input). Second, while AI systems can learn patterns in the data, explaining what drives those predictions or establishing causal inference is not a straightforward task (173). Moreover, cancer is a highly heterogeneous condition with multiple molecular, histological, and clinical subtypes that often respond differently to the same therapies (174). Therefore, ensuring generalizability of models across different cancer subtypes, treatments and patient populations may be an insurmountable task without access to vast amounts of accurately labeled data. Third, there is often significant delay in the timing between data collection, model training and the final model deployment. As a result, AI models are often outdated when deployed for clinical use, thus highlighting the need for more efficient pathways that would enable real-time updates. Finally, AI models are bias-prone often reproducing biases that are inherently present in the datasets used for training. Ensuring representation of diverse patient populations is of paramount importance to promote an equitable impact of AI in healthcare delivery and outcomes (175).

### Future Applications of AI in Cardio-Oncology and Molecular Imaging

With careful consideration of these limitations, AI has the potential to advance cardio-oncology in many different directions. Radiomic applications, which extract several metrics based on the shape, dimensions, signal density and spatial interrelationship of voxel signals in a given tissue, have been found to be superior to conventional readouts in reflecting tissue composition, as well as metabolic or inflammatory activity (176–178). In fact, some of the most exciting applications of AI lie beyond structural imaging in molecular imaging. In the recent past, deep learning and generative adversarial networks have successfully reconstructed PET images directly from raw sinogram data effectively maximizing image quality (179, 180). In other applications, AI tools have generated full-dose PET images from low-dose images, thus maximizing signal-to-noise ratio at lower radiation levels (181, 182). In another example, convolutional neural networks have enabled the development of cMRI virtual native imaging technologies which generate late gadolinium enhancement-like images in an accurate and reproducible manner without the need for contrast administration (183). Though originally developed in patients with hypertrophic cardiomyopathy, this technology may be of value in cardio-oncology and the monitoring of ICI-myocarditis. Further, for molecular imaging targeting biomarkers like FAP and PD1, these are already used clinically in oncology to monitor for residual disease and assess response to immunotherapy respectively, thus if the heart is captured in existing data sets, AI/ML can help to predict whether the presence of these markers are associated with adverse cardiovascular outcomes. Coupled with improvements in the speed and accuracy of segmentation algorithms, AI can accelerate the clinical deployment of molecular imaging approaches in the timely detection of cardiovascular toxicity (184).

## Conclusions

Imaging advances, particularly molecularly targeted imaging modalities may help detect cardiotoxicities at the earliest stages with greater specificity, shed light on mechanism as well as response to cardioprotective medications such as beta blockers, angiotensin converting enzyme inhibitors, etc. Newer MRI metabolic evaluation techniques such as hyperpolarized MRI may allow for a non-invasive approach to evaluate cardiac metabolism in real time. To complement imaging studies, use of AI and big data on imaging parameters and forthcoming molecular imaging datasets, in addition to patient demographics may help predict or detect cardiovascular toxicities at their earliest stages. Inclusion of diverse patient cohorts as well as cardiovascular parameters/biomarkers and imaging in cancer trials can enable AI/Ml to increase accurate categorization as well prediction models in cardio-oncology patients. Additional research in these areas and advancing animal studies toward human studies may further help improve cardiovascular outcomes in cancer patients.

## Author Contributions

JK led the development of the manuscript, writing, and generation of figures. EO contributed to writing and generation of figures. MH contributed to writing, assisting with editing and organizing of the manuscript. AJS oversaw the writing, editing, and review of the manuscript. All authors contributed to the article and approved the submitted version.

## Funding

This work was funded by NIH R01 HL113352, R01 HL121226, R01 HL123949, and R01 HL154345 associated with AJS.

## Conflict of Interest

AJS is a limited partner and consultant of MicroVide, LLC, which holds the license for the use of 99mTc-RP805 in myocardial applications. The authors declare that the research was conducted in the absence of any commercial or financial relationships that could be construed as a potential conflict of interest.

## Publisher's Note

All claims expressed in this article are solely those of the authors and do not necessarily represent those of their affiliated organizations, or those of the publisher, the editors and the reviewers. Any product that may be evaluated in this article, or claim that may be made by its manufacturer, is not guaranteed or endorsed by the publisher.

## Abbreviations

AI: artificial intelligence
CM: cardiomyopathy
CML: chronic myelogenous leukemia
DGE: delayed gadolinium enhancement
DNA: Deoxyribonucleic acid
ECV: extracellular volume
GLS: global longitudinal strain
ICI: checkpoint inhibitors
HER2: human epidermal growth factor receptor 2
HF: heart failure
HTN: hypertension
MI: myocardial infarction
MUGA: multigated acquisition
ROS: reactive oxygen species
TdP: torsades de pointe
TKI: tyrosine kinase inhibitor
VTE: venous thromboembolism.
